# Resilience effects in food consumption behaviour at the time of Covid-19: perspectives from Italy

**DOI:** 10.1016/j.heliyon.2020.e05676

**Published:** 2020-12-08

**Authors:** Carla Cavallo, Giovanna Sacchi, Valentina Carfora

**Affiliations:** a University of Naples Federico II, Department of Agricultural Sciences, 80055 Portici, Italy; b Free University of Bozen-Bolzano, Faculty of Science and Technology, 39100 Bolzano, Italy; c Catholic University of the Sacred Heart, Department of Psychology, 20123 Milan, Italy

**Keywords:** COVID-19, Lockdown consumption, Crisis food behaviour, Shelter effect, Comfort effect, Home meal preparation, Food science, Consumer sensory research, Qualitative research in food marketing, Agricultural science, Agricultural economics, Economics

## Abstract

The Covid-19 pandemic lead Italy to undertake a severe lockdown for almost two months. All of a sudden, the lives of Italians were forced to shift in accordance with the regulations issued by government. This change in the lives of Italians can be mirrored by an adjustment in food consumer behaviour that, consequently, brought about a transition in the whole supply chain. This paper gives an overview of the recent changes in consumption patterns that occurred due to the Italian lockdown, and how evolutions in behaviour are intertwined with the evolution of the main food supply chains. Many of the events here depicted are likely to last far beyond the crisis and affect the subsequent evolution of food consumption in Italy. The Italian retail supply chain successfully adapted to the big shift in consumption. Despite purchases for essential items having increased, no stockout harmed the food security of Italians. Out-of-home consumption moved inside houses giving space to home meal preparation and comfort food. Home delivery has been the most important element in this context, as it boomed during this period, helping laggard consumers fill the digital divide, as it was mostly mediated by e-commerce platforms and instant messaging. It was also the leverage that allowed small retailers and small producers regain their space. This crisis calls for a more sustainable food system that will be increasingly oriented to moving goods rather than people, which will also have relevance in the coming years.

## 1 Introduction

On the 31st of December 2019, the Municipal Health Commission of Wuhan (China) reported to the World Health Organization (WHO) a cluster of pneumonia cases of unknown disease in Wuhan City, in Hubei province. On the 9th of January 2020, the Chinese Centre for Disease Control and Prevention reported that a novel coronavirus (SARS-CoV-2) had been detected as the cause of the easily transmissible respiratory disease, later named Covid-19 (Ministry, 2020). On 11th March 2020, WHO declared the disease a pandemic (WHO, 2020). The spread of the infection has indeed been dramatic over the months recording an exponential increase: figures reached 9,206,609 cases and 474,730 corresponding deaths on the 23rd of June 2020, affecting 213 countries and territories around the world (Worldometer, 2020).

In the attempt to limit the outbreaks and contain the spread of the disease, many countries have adopted an emergency lockdown strategy. Lockdown measures have consisted of quarantines and restrictions such as borders closures, travel restrictions, limitations on personal freedom, closure of educational establishments, smart working at home (except for all strategic sectors), ban on public gatherings, social distancing, closure of all but essential commercial activities.

Amongst Western countries, the lockdown measures implemented in Italy have been considered one of the most restrictive closure schemes. On the 8th of March 2020, the Italian government imposed rigorous measures to its population, and the country became the first in the Western hemisphere to limit people to their homes. Leaving dwellings was possible only for a very limited range of activities such as food shopping, exercise, and dog walks, all within the vicinity of places of residence. This strict lockdown was maintained up to the 3rd of May 2020, after which citizens gradually were allowed to conduct their lives as normal, maintaining some minor precautions.

The purpose of the study is to shed light on the changes in the food paradigm, presenting an overview of the main effects of the Italian lockdown on consumption habits as well as on the corresponding psychological effects on consumers. In particular, the present study aimed to provide a categorization of the main changes in food purchasing, grouping them on the basis of underlying motives that may have guided Italian consumers during the lockdown. To do so, two theoretical backgrounds related to buying motives, respectively proposed by Babin et al. (1994) and by Nair (2004) have been applied. A motive is a need or desire that leads a person to act or behave to reach a desired endpoint. Product buying motives are those influences or considerations which provide the impulse to buy, induce action and determine choosing a particular product. Indeed, purchasing may be guided by both hedonic and utilitarian motives (Babin et al., 1994). Utilitarian motivations result from some type of conscious pursuit of an intended consequence and are directed toward satisfying a functional need (Babin et al., 1994). The hedonic motivations are instead based on searching for emotionally satisfying experiences. Similarly, product buying motives can be also divided on the basis of the nature of satisfaction sought by the buyer into emotional and rational dimensions (Nair, 2004). Emotional product buying motives include pride or prestige, emulation or imitation, affection, comfort or desire for comfort, sex appeal, ambition, desire for individuality, desire for recreation or pleasure, hunger and thirst and habit. Contrarily, rational product motives involve careful reasoning and logical analysis of the intended purchase. The buyer will work out whether it is worthwhile to purchase the product. Rational product motives are safety or security, low price, suitability, utility or versatility, durability, and convenience (Nair, 2004).

An analysis of the consequences of lockdown on different supply chains is also reported. In this framework, the paper provides an important opportunity to advance the understanding of changing consumer behaviour during an emergency crisis. Insights will help the comprehension on how to adapt and face the consequent changes by sales channels as well as different agri-food sectors.

Although it is not yet possible to provide a comprehensive review of changes in consumption behaviour at the time of Covid-19, it is hoped that the present analysis will pave the way for more research into the consumer behaviour field and serve as a starting point for marketing practitioners as well as policymakers.

The paper first focuses on data and method applied (section 2); it then gives an overview of the recent changes in consumption patterns due to the Italian lockdown (section 3); section 4 provides an analysis on the consequences of the lockdown for several supply chains. The fifth part is concerned with a discussion of the scenario previously depicted, while section 6 concludes with some early reflections on the change that this crisis initiated.

## 2 Data and method

All data used in the present study had been obtained by secondary data collection method merging information from the latest releases by food supply chains entities and sales data companies. In particular, information and data were collected from reports published from March to June 2020 by the following entities:

- General Confederation of Italian Agriculture – Confagricoltura;
- Italian Institute for Agricultural Food Market – ISMEA;
- Italian Federation of Food Industry – Federalimentare;
- The Italian Federation of Public Enterprises – FIPE;
- Nielsen Holdings plc.

Publications and reports from governmental institutions (Italian Ministry of Health), international organizations (World Health Organization) as well as press agencies (Italian Associated National Press Agency – ANSA) have been also considered in the development of the study. The accuracy of secondary data had been assessed through triangulation research.

The motivation of data adoption from existing sources lies in the unprecedented situation the pandemic has brought upon the whole society. Indeed, since the secondary data considered in the present study is associated with events that happened so few months ago, they represents the most updated source of information for making many early analyses and paving the way for more research as well as shedding light on the next adaptations needed at different supply chain levels. Several scholars, dealing with Covid-19 issue, have referred to such a source of information developing research on available data for outlining some early analyses (Aldaco et al., 2020; Arellana et al., 2020; De la Hoz-Restrepo et al., 2020; Sun et al., 2020). Therefore, secondary data analysis, allows for a much greater breadth of data across sectors and time, providing a larger and higher-quality baseline of information for analysing the unique event the world is currently experiencing.

## 3 Changes in consumption patterns

In 2019, figures reported that in Italy about 36% of meal consumption occurred out-of-home, for a total value of €86 billion (ISMEA, 2020b). In detail, approximately 10% of Italians had breakfast or lunch out every day, while more than 60% of Italians had at least one lunch or dinner a month outside of the home during weekends (FIPE, 2019)^1^.

As soon as the lockdown measures were undertaken, in a timespan of a few days, all consumption habits of Italians almost entirely moved inside the home. Consequently, large retailers supply chains quickly adapted to this shift, ensuring access to food for all groups of consumers, while other supply chains found it hard to adapt, as it will be subsequently explained in detail.

A substantial change in food consumption occurred according to the revolutionised daily routine of Italian consumers (constrained to stay at home and to reduce as much as possible the frequency of shopping occasions), but also psychological concerns arose. Due to all these circumstances, consumers slightly increased their purchases in supermarkets, resulting in a phenomenon that can be defined a resilience buying, rather than panic buying. As a main consequence, what happened is that each consumer bought a little more than usual in supermarket, due to the missed out-of-home meals and the reduction in shopping occasions.

**Figure 1:**
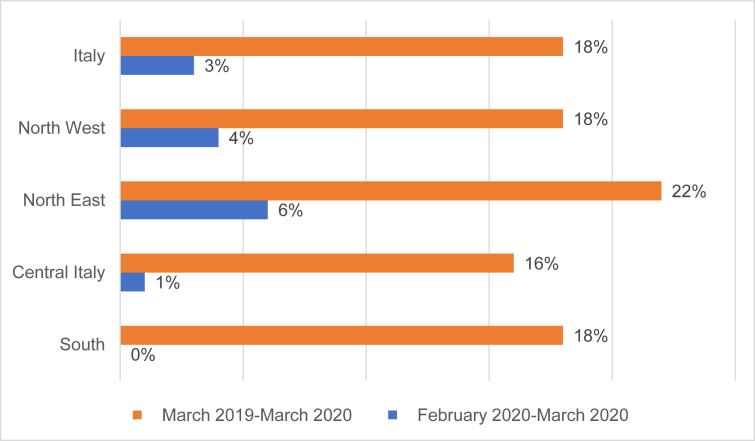
Annual and monthly variation in sales by geographical area∗ – 16–22 March 2019/16–22 March 2020–16–22 February 2020/16–22 March 2020. Source: (ISMEA, 2020a). ∗ According to Eurostat definition, Italian geographical areas are composed as follows: North West (Liguria, Lombardia, Piemonte, Valle d'Aosta); North East (Emilia-Romagna, Friuli-Venezia Giulia, Trentino-Alto Adige, Veneto); Central Italy (Lazio, Marche, Toscana; Umbria); South (Abruzzo, Basilicata, Calabria, Campania, Molise, Puglia).

Indeed, during the two months of lockdown in Italy, on average, total food retail sales increased by +18% compared to the previous year (ISMEA, 2020a; 2020c). Figure 1 shows the monthly (February 2020–March 2020) and annual (March 2019–March 2020) variations in sales by geographical area. The spatial distribution of increased sales shows that there is no direct connection between the geographical spread of the virus and the increase in food sales. Thus, it is possible to suppose that the behaviours changed according more to the perception of risk than the risk itself (Lobb et al., 2007). Furthermore, it is possible to suppose a saturation of cupboards especially in Southern and Central Italy, combined to the beginning of the liquidity crisis for some households. Indeed, although sales in those areas are +18% and +16% higher than in 2019, they stop growing in economic terms (0% and +1% respectively compared to the previous month). On the contrary, the North East shows greater dynamism in contrast to other areas, with a growing rate of +22% over the same period in 2019 and +6% compared to the previous four weeks (ISMEA, 2020a).

This variation trend has been shared variously among the different sales channel (Table 1). In particular, the e-commerce channel increased by +160%, despite the limited availability of stores of fulfilling the considerable consumers’ demand. It is interesting to note that the tendency to use e-commerce increased for all age groups, with peaks of +13% for over 65, who traditionally were the least familiar and trustful group of consumers (Di Mauro, 2020; Hobbs, 2020). Small shops also regained the attention of consumers, showing an increase in sales of +40% (ISMEA, 2020a; 2020c). This effect occurred for two main reasons: on the one hand, because of the presence of shops nearby residential areas, and, on the other, due to improvised deliveries organised by phone or via e-mail (Hobbs, 2020; RedazioneFood, 2020). While supermarkets increased their sales), discounts increased their sales by +15%. Conversely, hypermarkets lowered their sales by -3%, due to their distance from residential areas (ISMEA, 2020a).

**Table 1:** Variation in Italian sales channels after coronavirus crisis.

Sales Channels	Var. % 2019–2020
E-commerce	+160
Small shops	+40
Supermarkets	+44
Discounts	+20
Hypermarkets	-3

As far as the shopping basket is concerned, considering sales occurred from early March to mid-April 2020, it is possible to observe other changes which mirror the evolution of consumption motives among consumers (Hall et al., 2020). A recent study found that most of the participants had not changed the quantity of their daily meals (Di Renzo et al., 2020), so it is possible to assume that the change occurred in the quality of meals. In detail, the major shifts in consumption interested the place of consumption, as all meals were consumed inside the house. Also, more time has been dedicated to preparation, resulting in a different food experience linked to daily meals compared to what consumers were used to before the coronavirus crisis. Also, concerns have been raised for the healthiness and share of waste connected to the new food habits (Aldaco et al., 2020).

Variations in consumption patterns can be drawn according to three main routes of consumer behaviour. At first, consumers increased their buying products associated with “prevention”, cupboard products in particular, producing an effect which has been categorised as the *shelter* effect. Following this, the purchasing of *comfort* products ensued, and, in the end, a *MasterChef* effect has been recorded (Table 2). All these effects will be more extensively explained in the next sections.

**Table 2:** Consumption resilience effects during Italian lockdown.

Resilience Effects	Product	Quantity var. 2019–2020 (%)	€ var. 2019–2020 (%)
Shelter Effect	Oranges	+25	+36
	Yogurt and probiotics	+11	0
	Supplements	+17	NA
	Pasta	+66	+6
	Canned meat	+66	+6
	Canned tuna	+36	+5
	Cured meat	+32	+11
	UHT milk	+62	+1
Comfort effect	Popcorn	+90	NA
	Chamomile tea	+76	NA
	Crisps	+31	NA
	Confectionery spreads	+61	NA
	Wine	+18	+2
MasterChef effect	Yeast	+226	NA
	Flour	+213	-2
	Mascarpone	+99	NA
	Butter	+86	+2,5
	Eggs	+53	+1
	Canned tomatoes	+50	+8
	Cut-fresh vegetable	-20	-3

### 3.1 The shelter effect

The Covid-19 crisis led to a situation of health emergency that is likely to trigger an uncontrollable emotion of anxiety (Dammeyer, 2020). This in turn may lead consumers to preventive behaviours, such as concentrating all resources to store commodities, medicine, and food (Long and Khoi, 2020). Accordingly, in Italy a predictable change in behaviour occurred. Due to the need of lowering the frequency of shopping occasions, in fact, consumers increased their purchases of storable foods versus fresh food, and increased purchases of products with supposed health benefits. Actually, the planning horizons for grocery shopping have been substantially expanded (Cranfield, 2020), in order to acquire a sense of control over the crisis period (Loxton et al., 2020).

Data show that there were substantial increases in sales of products such as canned meat (+66%); canned tuna (+36%) and cured meat (+32.4%). Additionally, sales of UHT milk increased substantially, with peaks of +62%, consequently, sales of fresh milk in supermarkets did not increase, being incompatible with delayed purchase occasions. The sales of pasta peaked +66% compared to last year sales, even considering that pasta sales in other channels decreased, the overall quantity sold by pasta industry increased in absolute terms. For these products indeed, there has been a peak in sales in the first weeks of lockdown, when consumers quickly stockpiled staples, then this effect slowly weakened during April and the following weeks. In addition, the increased attention on health raised as a reaction to this event caused an increase in sales of products beneficial to health (Mollenkopf et al., 2020). In fact, oranges increased to peaks of +25% with retail prices skyrocketing up to 4€/kg compared to prices before the Covid-19 outbreak, which ranged from 1,30€/kg to 2,70€/kg (depending on variety and production process). Sales of yogurt and probiotics initially increased by +11%. In supermarkets, all types of supplements increased by +17%, with producers reporting that sales of some supplements doubled and also tripled, especially those related to the upper respiratory trait; but also others linked to several conditions induced by lockdown, such as anxiety, stress or sleep problems (Marzialetti, 2020). It is important to specify that the prevention is only perceived by consumers, as no remedy, up to date, exists for the novel coronavirus, as stated by Italy's Competition and Market Authority (AGCM) who warned that no preventive health claim can be used for any supplement or cosmetic product (Chu, 2020). Based on this evidence, this change in consumers' behaviours during the lockdown had been categorised as a “shelter effect”. The *shelter effect* could be traced back to a utilitarian motive, specifically the “safety and security” motive identified as a rational buying motive based on purchasing a product to take precautionary measures (Babin et al., 1994; Nair, 2004).

### 3.2 The comfort effect

A common eating pattern may be triggered by stressful events which can be defined as emotional eating. In this case, individuals tend to turn to food for psychological comfort rather than physiological needs (Jáuregui-Lobera and Montes-Martínez, 2020). This tendency is aimed at particular kinds of foods whose consumption evokes a psychologically comfortable and pleasurable state for a person, as the category of *comfort foods* (Wansink et al., 2003). Furthermore, sales data confirm that in the period of lockdown, the interest of consumers towards comfort food risen. In fact, the sales of popcorn (+89.9%) and crisps (+31%) increased, also causing some stock outs. Of course, also the sales of confectionery spreads increased substantially, reaching peaks of +61% compared to the same period of the last year. Moreover, sales of chamomile tea jumped of +76.3% compared to the last year, while sales of wine increased by +18.5%. Despite increases in these sectors, the loss of other sales channels entailed serious problems for the entire industry. This increase was not homogeneous for all categories of wine, in fact, sparkling wine decreased of -5.4%, while bag-in-box wine (usually very cheap) increased by +36.8% (ANSA, 2020b). The total sales of alcoholic beverages increased by +180%, also due to fake news claiming that alcohol might have preventative effects, to the point that the Italian National Institute of Health raised a concern for the worsening of alcohol addiction in Italy (ISS, 2020). The search for comfort foods, and the related “comfort effect” during the lockdown, could be traced back to a hedonic motive, specifically the emotional buying motives defined as “desire for comfort” (Babin et al., 1994; Nair, 2004).

### 3.3 The MasterChef effect

During lockdown, the kitchen has also had the role of being place of entertainment beyond a place of meal preparation (Di Renzo et al., 2020), this can be seen also as an emotional regulation strategy (Hamburg et al., 2014). The increased interest in cooking and baking may be defined as the “MasterChef effect”, which can be connected to a hedonic motive and an emotional motive of seeking to satisfy the desire for recreation or pleasure (Babin et al., 1994; Nair, 2004). This tendency is due also to a changed opportunity costs of time for most of the consumers, who rebuilt their daily routine (Cranfield, 2020; Zwanka and Buff, 2020). Accordingly, the consumption trends evolved following the route of collecting raw ingredients for home meal preparation. In fact, the only products for which there have been substantial stockouts at different points of sales all over the country have been flour (peaks at +212.7%) and yeast (peaks at +226.4%). Nevertheless, they are not essentials, so their lack of availability did not harm the food security of consumers. Increases also occurred for other recipes ingredients, such as eggs whose sales increased with peaks of +53%, butter +85.9%, mascarpone +99.5%, and canned tomatoes around +50%. Inversely, cut-fresh vegetable sales decreased around -20% (ISMEA, 2020a), as saving time suddenly was not a priority for consumers who preferred fresh vegetables due also to their longer preservability, in fact preservability became a priority due to the suggestion from the Government to shop for grocery as little times as possible.

**Figure 2:**
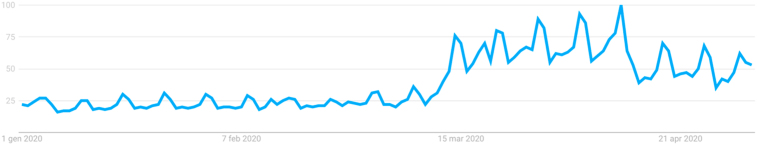
Google searches in Italy from 1^st^ January 2020 to 3^rd^ May 2020 for the word “recipe”. Source: trends.google.it, 2020.

Especially during weekends, as confirmed by Google Trends data, there was a substantial increase in searches on internet for the word “recipe” on the 9th March 2020, with weekly peaks on Saturdays (Figure 2). This effect was much stronger in the first weeks of lockdown, then gradually decreased. The detail of searches shows that the top-five recipes searched on the internet were: 1) pizza; 2) bread; 3) pancake; 4) custard; and 5) pie. This explains the increased request for flour and yeast that occurred during this period. Indeed, a large proportion of food players quickly leveraged the growing interest toward cooking from consumers, building proper marketing campaigns centred around the cooking experience to trigger an affective response toward the product.

## 4 Consequences for different supply chains

This period of lockdown resulted in different problems for the different supply chains of the Italian agri-food sector. On the one hand, it is possible to highlight the resiliency of the sector in providing stable access to food but, on the other hand, the fragility of the supply chain has also been revealed (Mollenkopf et al., 2020). The closing of spaces that support social activities had different repercussions on supply chain actors. Specifically, producers that had ho. re.ca. as the exclusive sale channel dramatically suffered from this situation (it has been estimated a -40% decrease of out-of-home consumption), while others who quickly redesigned their sale channels, organized deliveries, or had multiple channels were less vulnerable. Despite the quick increase in demand for food through supermarkets, several categories of agri-food found it hard to place their products on the market.

The most important restriction was aimed at reducing the movement of people, therefore agritourism and rural tourism were completely suspended. They represent an important share of revenue created through agriculture-related activities (about 30%), but also, they serve as a marketing channel for tourists from abroad, especially for Italian high-end products. As a result, these have been the ones that suffered the most from this crisis.

Particular is the case of wine, as it is composed of different categories of products that have their own sale channel and target market. As said before, sales in supermarkets definitely increased, but they may not have covered the losses of other channels. In addition, sales through e-commerce skyrocketed, as testified by Tannico, one of the greatest online platforms for wine sales, which had an increase in sales of about +100%. However, the consumption patterns shifted towards bigger size and cheaper products (Rusconi, 2020).

Another source of difficulty has been represented by the loss of demand from abroad, with the ‘lockdowns’ in other countries decreasing international consumers' demand for Italian wines, and entailed an expected loss of around €1 billion of exported products (L.Ben., 2020). The next harvesting period will bring to light issues of storage, due to the decreased demand resulting from the pandemic. To this purpose, together with the shortages of sanitizing products, wineries began to distil sanitizing alcohol. Since this process is way more expensive than distillation from cereals, the government allocated €50 million in subsidies, to sell it to consumers at affordable prices and make room for new wine (ANSA, 2020e).

The Italian fishery industry also lost a substantial part of their revenues during this period, being indeed the industry that mostly suffered from this crisis period (De Santis, 2020). Considering just March 2020, 70% of fishery turnover, amounting to €60 million, has been lost, with a negative impact of -6% on the yearly trend (ANSA, 2020c). In fact, ho. re. ca. channel, which holds about 55% of the fishery market share (Nielsen, 2020a), has totally disappeared, with particular reference to recreational fishing which holds 15% of the market share. Furthermore, on the export side a negative impact has been recorded, amounting for -20% compared to 2019 (Confagricoltura, 2020). Additional difficulties for the industry rely both in partial fishing prohibitions, both in the closure of fish markets during this period (ANSA, 2020d), so, despite being a demand for these products from consumers, it has not been possible to find a market for them, and consumers oriented their purchasing towards frozen products.

Other problems are still in line for the livestock sector that is slowly adapting to the change in the situation for markets. For example, a substantial part of fresh milk lost its market with the closing of cafes and ice-cream bars (-25%), furthermore, consumers preferred types of milk with a longer shelf-life, as UHT, due to the abovementioned *shelter effect*. Therefore, part of the losses have been covered through the transforming of fresh in UHT milk, but there are still difficulties for the sector (Borrillo, 2020).

For the bovine meat sector, the most important problem relies in the lack of balance for meat cuts sales. In detail, high-end cuts are sold abroad or at restaurants, while the rest of the meat is sold through supermarkets. Thus now, just a part of these cuts is regularly sold and consumed, while there is still an excess of meat that has no market. This caused a decrease in price, which in turn led to a reduce in slaughterhouses requests for meat (ISMEA, 2020b).

While, in the case of pork meat, there are no such problems of sale channel as almost all the meat products are sold through supermarkets, however production did decrease due to distancing measures in the production structures (ISMEA, 2020b). Despite the demand for cured meats having increased in the supermarkets, as explained in the previous paragraphs, the sector lost about 25% of the markets with the closing of restaurants, so total sales are lowered. This is a limited problem due to the long storage possible for these products.

## 5 Discussion

Based on recent events that characterized food sector, it is possible to draw some preliminary insights. The first finding is that, globally, the Italian organized retailers supply chain successfully adapted to the shift in consumption due to the Covid-19 pandemic emergence. While, for instance, in the US there has been a rush in stockpiling essentials that emptied groceries of several products (such as milk, meat, and toilet paper) (Kirk and Rifkin, 2020), to the point that the American President was asked to reassure the population about the future availability of products (Lapin, 2020). Also in China several cases of panic buying have been highlighted (Wang et al., 2020). None of these occurred in Italy, despite a few cases of panic grocery, occurred sporadically only in Milan area (ANSA, 2020a), supermarket shelves have been kept constantly filled and few stockouts occurred (Federalimentare, 2020). In fact, no difference has been found in consumer behaviour between areas hit by the virus and other parts of the country. Stockouts occurred only for flour and yeast that are not essentials, therefore the consumers were always provided with the goods they needed. Conversely, stock-outs represent a problem, as due to herd mentality they lead to further stockpiling (Cranfield, 2020; Loxton et al., 2020). In Italy, the rare presence of stockpiling behaviours might be explained by the social trust in institutions, which is a relevant factor in influencing consumers' food choices (Carfora et al., 2019; Del Giudice, Cavallo and Vecchio, 2018). Stockpiling behaviour is a panic buying behaviour definable as buying an unusually large number of products to avoid possible future shortages and reduce a feeling of insecurity (Shou et al., 2013). Several studies have shown that trust in institutions can buffer the effects of risk perception on consumers’ behaviour (Cembalo et al., 2019). Thus, probably the Italian consumers took comfort in reassurance from institutions, consequently reducing their perception of risk from the possible shortage of food products and the consequent stockpiling behaviours.

The most remarkable - as well as predictable - consequence that has been observed in Italy is related to the type of food purchased. Indeed, most of the consumers kept their usual number of meals, but within a matter of days, they changed the sale channel and used food as a comfort and as entertainment. This is confirmed by other studies which found that buying more indulgent and comfort food also characterised the consumer behaviour of other countries as Spain (Laguna et al., 2020). A concern has also been raised toward an increased risk of obesity triggered by different factors: people consumed more comfort food, physical activity has been reduced due to restrictions in people's movement (Mumena, 2020; Nouh et al., 2020). But also other events put at risk consumers' health, for example the shut-down of school catering exposed children to unhealthy food, especially for lower income consumers (Rundle et al., 2020). This trend is believed to go on after the crisis due to the foreseeable recession that will harm the possibility of several groups of consumers to afford healthy food (Aldaco et al., 2020).

It can be presumed that the shift of consumption from out-of-home to home can last also after the pandemic crisis. In fact, an economic crisis is expected as a result of a substantial reduction in both consumption and production of goods and services, and in every economic crisis out-of-home consumption lowers (O'Dare Wilson and Scott, 2015). Actually, there has already been a recent increase in sales of the discount channel in the post-quarantine time (ISMEA, 2020b), this trend is believed to characterize the post-crisis years (Aldaco et al., 2020). Then also makes out-of-home consumption lose its appeal of entertainment due to all social distancing rules and queues. Furthermore, smart working is still encouraged, and this lowers the share of workers who have their lunches in workplaces catering facilities, and restaurants close to the place of work.

As for the sales channels, food e-commerce recorded the most impressive increase, tripling online purchasing compared to the figures registered the previous year (ISMEA, 2020a; 2020b; 2020c). This trend is supposed to last long after the health crisis, due to the familiarity that consumers have built with this sale channel that was way lower before the pandemic crisis. This phenomenon “helped” to fill the digital divide gap also in the retail online of food products (Nielsen, 2020a). During lockdown, the use of new technologies dramatically increased for all age cohorts, this increased familiarity with delivery services mediated by online services and apps both from consumers and producers and restaurants (Food Service, 2020a). Online services also mediated the sales made by short food supply chains (e-commerce and instant messaging), which dynamically constituted micro-networks which supplied food in a complementary or alternative to usual way (Calori and Federici, 2020). This helped to foster a digital transformation also for small farms (Butu et al., 2020). Short supply chains had an essential role in enforcing the resilience of food networks in contributing to the stability of access to food also in crisis periods (Béné, 2020; de Paulo Farias and de Araújo, 2020). Since barriers to online shopping were broken down for a substantial part of consumers, also the most resistance to change, the adoption curve for e-commerce has been accelerated by the crisis and it can be presumed that this evolution will last long after the Covid-19 crisis (Hobbs, 2020).

In a sense, the Covid-19 emergency has been an accelerator of processes already underway. The model of hypermarkets, a massive distribution structure developed in the 1950s in the United States – which presents a very different urban planning to the Italian one – has proved unsuccessful and requires its overcoming. On the other hand, small-scale local shops have demonstrated the ability to easily adapt to the new situation and to be a valuable business model to overcome the problems posed by the lockdown. Furthermore, the owners of neighbourhood shops enjoy a human relationship with their customers and during the lockdown they have guaranteed home deliveries to the elderly and most vulnerable people, understanding that valuing these kinds of services and investing in e-commerce, increase their chances of survival.

Remarkable is the role of food that went far beyond the simple nutritional provision. The shelter effect suggests that in a situation of emergency, staple foods regain their importance in the food basket and this partially inverted the tendency of consumers in increasingly differentiating their food purchases (Marinelli et al., 2015). While the comfort effect suggests that the role of food as an emotional regulator is very strong, and this can shift consumers’ behaviour in particular moments of their life. These tendencies partially shadowed vegetarian and healthy food preferences that were largely expanding before the crisis (Sadler, 2004; Ueland et al., 2020).

Then, behind the success of cooking and baking, there has been not only time availability, but also some psychological motives. Given that during the lockdown period people reported both an increase in negative and a decrease in positive affect (Aymerich-Franch, 2020), the notable increase in cooking and baking could have been used as an emotional regulation strategy. Generally, cooking favourite recipes encourages the retrieval of emotions and experiences from the past, which involves an inter-related web of associations across space and time including people, places, and events (Baker et al., 2005). In addition, food offering is recognised as a means to positively affect both the provider and recipient (Hamburg et al., 2014). Based on these assumptions, during the lockdown cooking food could had two functions. On the one hand, this activity probably helped lonely people to overcome social isolation and negative emotions. On the other hand, in the family context, preparing food aimed to attenuate both the food provider's negative emotions and those of other family members.

Then, considering Italian supply chains, the evidence is that industries and producers who had differentiated sale channels and sourcing had a better chance of survival through directing all produce towards organized retailers (Béné, 2020), while there have been significant difficulties for the ones who only relied on a single channel or customer, as it is the case of companies which supply only to ho. re.ca. or exclusively to customers abroad for their sales. This is the case of Italian fisheries, who suffered the most from this crisis. For them, delivery has been allowed to cope with markets closure, but it has not been sufficient to avoid a substantial loss of value. Therefore, the demand of consumers has not been fulfilled and they substituted fresh fish products with frozen products.

Finally, a survival strategy for small producers has been represented by new technologies. In fact, small farms have been able to organize quickly a delivery service through phone and instant messaging that helped to change their business model quickly and without any big investments that would be necessary for a proper e-commerce platform. This represents a valuable source of innovation at the hand of all small farms, but also it has been leveraged by small local shops.

## 6 Conclusion

In 2020, due to the Covid-19 health emergency and the consequent lockdown, Italian out-of-home food consumption fell by -40% with an estimated loss of €34 billion, compared to 2019 when the total Italian household out-of-home spending was nearly €86 billion. However, according to ISMEA (2020b), out-of-home consumption loss is somehow balanced by the increase in retail sales, which makes it possible to estimate an increase in domestic consumption of about +6% compared to 2019, when the total Italian household domestic spending was nearly €163 billion. Given these assumptions, in 2020, the overall impact on total domestic and out-of-home agri-food expenditure would decrease of about -10%, amounting to around €24 billion (ISMEA, 2020c). To these figures, it has to be considered also the impact on household expenditure capacity caused by the economic crisis which will follow the health one according to several observers.

As shown, an important change in the consumption paradigm occurred, with behavioural changes either due to a completely different daily routine of Italians during the lockdown period, or because food helped to cope with the stress and anxiety feelings that were triggered by the health crisis, or for a change in people's time opportunity cost. Food, once again, mirrored the profound change of Italian society that arose in this period. Indeed, it represented an emotional regulation source, a source of entertainment, and it showed the trust that Italian consumers have towards food supply chains. This evolution is only in an early phase, considering that behaviours and routines were completely changed in a very limited timespan, and the near future holds a health and economic crisis period as never before experienced. Therefore, food actors need to adapt to a new consumer behaviour that has to take into account changes triggered by new daily habits and new needs and shopping motives.

In sum, food supply faced several challenges, but the sector showed a high resiliency succeeding in the provision of a stable access to food also to lower income consumers. Some actors, especially the ones which had a low level of differentiation in sourcing and output channels, suffered the most from anti-coronavirus measures and lockdown, therefore some policy actions are needed in this sense.

From a managerial point of view, the phenomena such as the dramatic increase in e-commerce, and the significant repositioning of proximity farming and retailing, established. These innovations in daily purchasing behaviours can be interpreted as a result of socio-ecological resilience, which is related to the opportunities that a disturbance opens in terms of the reorganizing of structures and processes, the renewal of the system and the emergence of new trajectories (Folke, 2006). Thus, it is possible to presume that these trends may be long lasting after the crisis. This suggests the strengthening of a new relationship of trust between the inhabitants and the local sales network.

These aspects open the discussion on the possibility of moving towards more sustainable and convenient economic and social models - for humankind and the environment as a whole. It is indeed true that the e-commerce channel has been a strategic tool in dealing with consumers’ food procurement during the lockdown. Nevertheless, a viable national strategy should look at a new distribution paradigm that can improve quality of life and meet market needs. In the new “normality” of distribution, indeed, it will still be better to move goods rather than people, and for this reason those issues linked to the last mile logistic and to viable delivery models have to be tackled to rethink an economic model in line with ecosystem sustainability (Kotler, 2020).

## Declarations

### Author contribution statement

G. Sacchi and C. Cavallo: Conceived and designed the experiments; Analyzed and interpreted the data; Contributed reagents, materials, analysis tools or data; Wrote the paper.

V. Carfora: Analyzed and interpreted the data; Contributed reagents, materials, analysis tools or data; Wrote the paper.

### Funding statement

This work was supported by the Open Access Publishing Fund of the 10.13039/501100008815Free University of Bozen-Bolzano.

### Data availability statement

Data included in article/supplementary material/referenced in article.

### Declaration of interests statement

The authors declare no conflict of interest.

### Additional information

No additional information is available for this paper.

## Footnotes

1 Some restaurants tried to deal with the lockdown measures by organising home delivery, which was possible in many regions of Italy, while in other regions it was forbidden (i.e. in Campania and Sicily). However, data state that, among traditional restaurants, only 5% was already providing this service, while 10% organised deliveries because of the lockdown (FIPE, 2020). The remaining 85% of restaurant managers affirmed that home delivery was not an option for their business for organizational reasons (FIPE, 2020). Nevertheless, it is relevant to underline that in 2019, the home delivery market in Italy had a value of €590 million, corresponding to 1% of out-of-home consumption (Food Service, 2020b). Thus, this channel cannot be considered central in the ho.re.ca. sector in Italy and cannot be able to cope for missed revenues of restaurants.
